# Adipose and Muscle Cell Co-Culture System: A Novel In Vitro Tool to Mimic the In Vivo Cellular Environment

**DOI:** 10.3390/biology10010006

**Published:** 2020-12-24

**Authors:** Palaniselvam Kuppusamy, Dahye Kim, Ilavenil Soundharrajan, Inho Hwang, Ki Choon Choi

**Affiliations:** 1 Grassland and Forage Division, National Institute of Animal Science, Rural Development Administration, Cheonan 330-801, Korea; kpalaselvam@korea.kr (P.K.); ilavenil@korea.kr (I.S.); 2 Faculty of Biotechnology, College of Applied Life Science, Jeju National University, Jeju 63243, Korea; pioioiq10@gmail.com; 3 Department of Animal Science, College of Agricultural and Life Science, Jeonbuk National University, Jeonju 54896, Korea; inho.hwang@jbnu.ac.kr

**Keywords:** co-culture, in vitro technique, adipocytes, muscle, secretory factors

## Abstract

**Simple Summary:**

Co-culture system provides a novel platform to study interaction between different cell types in an in-vitro method. The co-cultures techniques have played key role in the understanding of cell–cell communication and relevant for drug response analysis. Co-culture system could influence therapeutic drug response in a dose dependent manner which reflects the clinical situation in patients. Also, the co-culture system may reflect a more realistic environment that similar phenotypic and functional characteristics of in vivo conditions. We also suggest that the co-culture methods as a key tool to study the interaction between adipose and muscle tissue under various environments including drug responses, production and influence of secretory factors, cell growth, and development. Therefore, the co-cultures method has been shown wide applications in cell biology study.

**Abstract:**

A co-culture system allows researchers to investigate the complex interactions between two cell types under various environments, such as those that promote differentiation and growth as well as those that mimic healthy and diseased states, in vitro. In this paper, we review the most common co-culture systems for myocytes and adipocytes. The in vitro techniques mimic the in vivo environment and are used to investigate the causal relationships between different cell lines. Here, we briefly discuss mono-culture and co-culture cell systems and their applicability to the study of communication between two or more cell types, including adipocytes and myocytes. Also, we provide details about the different types of co-culture systems and their applicability to the study of metabolic disease, drug development, and the role of secretory factors in cell signaling cascades. Therefore, this review provides details about the co-culture systems used to study the complex interactions between adipose and muscle cells in various environments, such as those that promote cell differentiation and growth and those used for drug development.

## 1. Introduction

In vitro co-culture techniques can be used to mimic in vivo environments and to observe interactions among cells (autocrine) and between cells (paracrine) [1]. Co-culture systems may be used to explore the mechanisms of action of drugs and their potential targets; they help to bridge the gap between mono-culture methods and animal models. Co-culture systems can be divided into two main categories, indirect methods and direct methods [2]. In the indirect methods, cells are physically separated into two different populations using trans-well inserts and/or overflow culture chambers that allow communication only via secretory factors. On the other hand, direct co-culture methods allow cell–cell interactions between different type of cells, which is typically achieved by spatially controlling the positions of adherent cells within a culture dish [3]. Generally, co-culture systems are used to study the secretory and transcription factors which are partially/fully involved in induction of cell differentiation, regulation of cellular proliferation, and production of metabolites for signaling cascades. A co-culture system can be used to reduce the amount of drug needed for a study, identify the target organs of a drug, and predict the adverse effects of drug metabolites [4]. Thus, this technique shows great potential for cell toxicological studies in the future and is now used chiefly for pharmacodynamic research.

**Figure 1:**
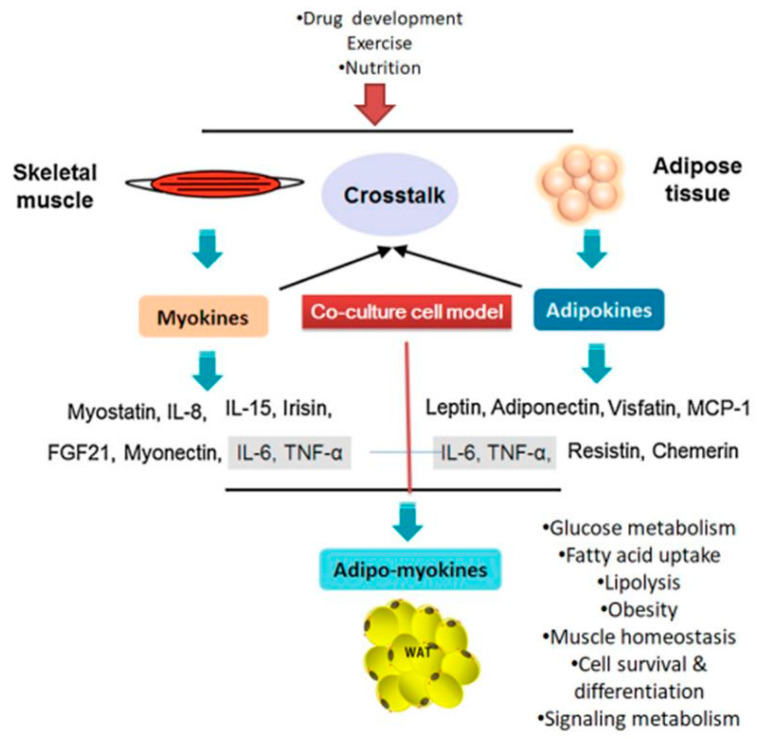
Cross-talk between muscle and adipose tissues is regulated by various secretory factors (adapted and modified from Li et al. (2017) [14]). WAT: white adipose tissue; TNF-α- tumor necrosis growth factor- alpha; FGF21: fibroblast growth factor 21; IL: interleukin.

Co-culture systems are widely used to study cross-talk between different cell lines, including adipocytes, endothelial cells, fibroblasts, macrophages, and muscle cells. Also, it is a crucial tool for understanding the various metabolic connections between adipose and other tissues [5]. The co-culture studies can provide realistic insights on cell–cell interactions via secretory factors that are effect in various metabolic functions such as energy homeostasis, muscle atrophy, and obesity and related co-morbidities. Previously, Ruiz-Ojeda et al. (2016) [6] investigated the co-culture relationship between adipocytes and macrophages and how they communicate under conditions that mimic obesity, insulin resistance, or inflammation. Similarly, an indirect co-culture system used to study intercellular communication between muscle and adipocyte cells was developed by Choi et al. (2013) [7]. Pre-adipocyte differentiation is regulated by differentiation myoblasts in the co-culture system, while pre-adipocytes promote adipogenic gene expression in muscle satellite cells co-cultured with pre-adipocytes. Muscle and fat tissue are major paracrine and endocrine organs that communicate with each other regarding muscle development, regulation of energy homeostasis, and insulin sensitivity [8]. For example, exercise-induced improvements in muscle function influence carbohydrate and fatty acid metabolism in the whole body as well as peripheral insulin sensitivity. Skeletal muscle communicates with other tissue types (i.e., adipose) to regulate, either directly or indirectly, whole-body energy homeostasis through myokine release [9] (Figure 1). Muscle-derived secretory proteins—including IL-6, irisin, myostatin—and some peptides, known as myokines, regulate adipogenesis via paracrine and endocrine effects [10]. Recently, Chu et al. (2016) [11] reported that porcine pre-adipocyte differentiation was inhibited in a C2C12 co-culture cells and that the expression levels of early differentiation marker genes in adipocytes were lower than those in mono-cultured adipocyte cells. Recently, Shahin-Shamsabadi [12] developed a 3D bio-fabrication method using adipocytes and myoblasts, that analyzed specifically either in direct physical contact or in close proximity such that the paracrine interaction between the cells. The physical contact between cells have been flouted in co-culture systems using transwell inserts and can be used in studies for the development of anti-obesity drugs. Anan et al. [13] studied the method for analyzing the direct interaction between adipose tissue and cardiomyocytes. The HL-1 cells suppressed the development of CD44+/CD105+ mesenchymal stem cell-like cells and lipid-laden preadipocytes from ATFs. In addition, the HL-1 cells stimulated the secretion of adiponectin in adipose tissue fragments (ATF), whereas they decreased production of leptin in a co-culture experiment.

## 2. Adipocytes/Muscle Cells Co-Culture Models

Co-culture models have been used to examine diverse cellular functions, such as interactions between muscle and nerve cells, angiogenesis, adipocyte/muscle cell cross-talk, immune cell functions, etc. [15]. Interactions between co-cultured myoblasts and adipocytes have been implicated in facilitation of muscle growth, tissue repair and muscle regeneration. These findings have led to the discovery of adipo-myokine secretory factors (AMSFs), which are produced by adipocytes and myocytes to induce differentiation and proliferation. Table 1 summarizes the findings of recent studies in co-culture systems using adipose and muscle cells.

At present, our knowledge of the interactions between adipocytes and myocytes largely stems from studies of the effects of individual adipokine factors on cultured muscle cells or adipocytes and vice versa. A wide variety of adipocyte mediated secretory molecules regulate muscle metabolism without affecting other tissues. Furthermore, in an in vitro co-culture system, myocytes were exposed to a group of free fatty acids (FFAs) and adipo-myokines [16,17] that communicate signals to other organs. These studies have fueled attention towards analyses of metabolic communication between fat and muscle cells. Adipose tissue is known to protect other cell types from lipotoxicity by providing a safe place to store surplus energy. However, obesity-related dysregulation of adipose tissue promotes lipid oversupply to several non-adipocyte tissue types. This can contribute to the development of metabolic diseases, such as cardiovascular disease and liver and bone disorders. Adipose tissue is an important endocrine organ that communicates with the brain and peripheral tissues to bring about changes in whole-body energy homeostasis through a network of circulating adipokines [18]. These signaling factors include peptide hormones; chemokines such as leptin, adiponectin, resistin, visfatin, and apelin; and pro-inflammatory cytokines including interleukins (IL-1β, IL-6 and IL-15) and tumor necrosis factor-α (TNF-α). Obesity may lower the levels of circulating insulin-sensitizing adipokines such as adiponectin while increasing the levels of pro-inflammatory response molecules such as IL-6 and TNF-α in adipose tissues [19].

**Table 1:** Examples of co-culture model systems consisted of adipose and muscle cells.

Co-Culture Model	Compounds Used	Study Findings	Ref.
Pre-adipocytes-myoblasts	Arginine and/or trans 10, cis-12 CLA	Increased adipogenic gene expression in myoblasts	[7]
Bovine adipocytes and pre-adipocytes	Adipogenic induction medium	Increase lipolytic response and glycerol release	[20]
3T3-L1 adipocyte–C2C12 cells	Ferulic acid	Increase lipolytic profile and glycerol release	[21]
C2C12 myoblasts-3T3-L1 adipocytes	Adipocytes medium induced IL-6	Suppress the differentiation of C2C12 cells	[22]
Differentiated C2C12 with 3T3-L1 cells	Calcitriol	Decreased anti-inflammatory cytokines production	[23]
3T3-L1 (adipocyte)-L6 muscle cell line	Differentiation media without additives	Co-culture adipocyte cells increased GPDH activity	[24]
Human fat and skeletal muscle cells	Differentiation medium with 1 pmol/L insulin	adipocyte induce a paracrine perturbation in muscle cells	[25]
3T3-L1 preadipocytes-differentiated C2C12	DMEM differentiation medium	C2C12 suppressed the mRNA, protein expression of glucocorticoids receptor	[11]
C2C12 myocytes and 3T3-L1 adipocytes	Adipocyte conditioned medium with Leucine	Modulation of muscle and adipocyte energy metabolism	[26]
C2C12 myocytes and 3T3-L1 pre-adipocytes	Zinc oxide nanoparticles	Increased expression of antioxidant enzymes and mRNA expression	[27]
3T3-L1 adipocytes with RAW 264 macrophage	Dietary calcium	Reduce the inflammatory cytokine and oxidative stress in adipocytes	[28]
3T3-L1 adipocytes with splenocytes cells	Lipopolysaccharides (LPS)	Elevated cytokine secretion (TNF-a, IL-6, MCP-1)	[29]
Murine adipocytes-C2C12 cells	Leucine and calcitriol	Decrease energy storage in adipocytes and increasing fatty acid utilization in C2C12	[30]
3T3-L1 pre-adipocytes and C2C12 muscle cells	DMEM/FBS growth medium	Promote the mitochondrial biogenesis bydirect activation of SIRT1 in both cells	[31]
3T3-L1 pre-adipocytes and L6 muscle cells	DMEM/F12 supplemented with BSA	Oxygen species production and level of Glut1 mRNA and protein increased in L6 cells	[32]
Primary human adipocyte and skeletal myotubes	Low serum differentiation medium	Understating the metabolic function of intra muscular adipogenesis (lipolytic activity)	[17]
3T3-L1 cells with J-6 cells	Defined Medium for co-cultured cells	Low level of IGF-1 IGF-II are not likely to play a role in intercellular communication between these cells	[1]
Porcine pre-adipocytes and muscle satellite cells	DMEM/F12 medium	Induce cell growth and proliferation meanwhile, inhibited the cell differentiation	[33]
Skeletal muscle (L6)-adipocyte (3T3-L1)	Specific differentiation medium for both cells	IL-6 cytokine plays main role in cross-talk between these cells	[34]
3T3-L1 and L6 cell line	Differentiation medium containing 5% HS	Adipocyte differentiation inhibited and suppress the lipogenic gene expression	[35]

## 3. Monoculture vs. Co-Culture

### 3.1. Monoculture Techniques

Cell culture techniques permit us to understand development-related diseases, drug activity, secretory protein profiles, and different types of cell–cell interactions. These techniques are mainly used to evaluate the preliminary level of drug toxicity in in vitro disease models, and can be used to identify gene function in the laboratory environment [36]. Though cell culture models are very common, they are limited in their ability to represent complex in vivo environments, and the results may not be relevant in certain cases [37]. Co-culture models more accurately represent the natural environment. Also, single cell culture methods are most commonly used to grow a single type of cell, but recently 2D and 3D culture methods have gained popularity due to their diverse biomedical and clinical applications [38].

### 3.2. Co-Culture Techniques

Co-cultures models are highly applicable to drug development research as they offer a more in vivo-like tissue model without the complications associated with animal models. The cellular growth and differentiation mechanisms in a co-culture system may differ from those in a mono-culture system [39]. Thus, it is essential to study the mechanisms of cellular cross-talk between different cell types in co-culture systems [40]. Furthermore, some cells are not easy to grow in in vitro mono-culture systems and will not exhibit preferred in vivo physiological behaviors [41], but may be successfully co-cultured or exhibit the behavior of choice in a co-culture system.

Studies of co-culture-related phenomena are generally kept as simple as possible. For instance, co-cultured muscle satellite cells were analyzed by determining variations in cell number, morphological alterations, and the number of cells that differentiated into multinucleated myotubes [1]. Similarly, pre-adipocyte status in co-culture systems has been assessed mainly by determining cell growth and morphological changes. Recently, the importance of complex interactions between muscle and adipose cells has been understood in regard to the pathogenesis of non-communicable metabolic diseases. Adipocytes are present at different sites (abdomen, hip, thigh, etc.) that are inhomogeneous and differ quite considerably in their metabolic and inflammatory functions, and can be clearly differentiated in part by adipose depot differences. Hence, use of co-culture systems could advance our understanding of the types of interactions between muscle and fat tissues and other organs. Also, this system provides a more physiologically accurate picture with which to examine the role of secreted factors in governing cell–cell interactions [42].

Dietze et al. (2002) [25] reported that co-culture of human myocytes and adipocytes enhanced adipose-derived secretory factor signaling in cross-talk with skeletal muscle cells, mainly insulin-stimulated phosphorylation of protein kinase B (Akt) and insulin receptor substrate 1 (IRS-1) in myocytes. There is sound evidence at the phenotypic and cellular levels that adipose-mediated secretory factors interfere with muscle insulin signaling and homeostasis [43]. Endocrine cross-talk between whole fat tissue and whole skeletal muscle have yet to be examined ex vivo. Furthermore, Tishinsky et al. (2014) [44] studied the effects on dietary fatty acid consumption on regulation of adipose tissue-skeletal muscle cross-talk in a co-culture system. The study found that dietary fatty acid intake regulates excess adipose fat depots. Similarly, Bruckbauer and Zemel (2011) [31] reported on calcitriol and leucine modulation of sirtuin 1 (SIRT1) in adipose tissue and skeletal muscle, and found that SIRT1 is the central signaling target that mediates the effects of calcitriol and leucine. In sum, adiposity is highly related with changes in glucose and fatty acid metabolism.

## 4. Co-Culture System Advantages and Disadvantages

Co-culture systems are used to culture and differentiate cells in vitro and are of great importance to the process of the drug development as well as treatment of incurable pathologies [45]. In vitro monoculture models are commonly used to study complex mechanistic aspects of drug response and the paracrine effects of drugs. However, co-culture techniques may provide a simplified, more cost-effective, high-throughput technique that utilizes fewer animals and allows for more focused analysis.

However, as this is a somewhat simplistic approach, these in vitro cell culture systems may produce inaccurate results; they may, for example, examine an inadequate number of variables and not include different types of cells and their responses that would be necessary to authentically duplicate in vivo aspects of the foreign body response (FBR). As a result, in vitro models may not be able to predict certain in vivo phenotypes. For instance, a major contradiction between in vitro and in vivo models is the observed lack of inflammatory stimulation in vitro [46].

2D cell culture has many advantages, e.g., it is relatively simple and easy to handle cells in vitro and to perform different functional tests. Cellular interactions are responsible for cell proliferation, differentiation, expression of genes and proteins, responsiveness to stimuli, drug responses and other cellular metabolic functions [47,48]. Also, changes in the structure of cells can affect their function and metabolism [49]. Moreover, cells in a monolayer have unlimited access to the components of the medium—such as O_2_, nutrients, metabolites, and signaling molecules—which is not generally the case in 3D systems.

One problem of co-culture systems concerns the many variables, including the composition of the medium, volume, and duration of culture period and the degree of similarity and separation between two different cell populations, which need to be optimized [50].

The animal models are used to study the molecular mechanisms of development and progression of diabetes and cancer and metabolic diseases. However, the transgenic animal’s experiments are expensive, difficult to visualize, and they are not completely demonstrative of human physiology or genetics. In addition, it needs to get associated ethical clearance [51].

## 5. Secreted Factors in Co-Culture Model

Growth factors are biochemical signals that are naturally produced by cells/tissues and are responsible for cell growth, development and repair. They include fibroblast growth factors (FGFs), insulin-like growth factor-1 (IGF-1), β-nerve growth factor (β-NGF), transforming growth factor-β (TGFβ), etc. For example, the L6 cells were co-cultured with 3T3-L1 cells for 24 h, followed by their stimulation with insulin (100 ng/mL) showed increases Akt phosphorylation at both sites (ser473 and ser308) in adipocytes. However, these effects were partially inhibited by 3T3-L1 co-culture cells [32]. Therefore, the co-culture system can be potent way to study cell signaling between two different cells like in vivo model.

Cytokines are a diverse group of secretory substances that play specific roles in the interactions and/or communications between two or more different type of cells. They include interleukins (ILs), tumor necrosis factor, etc. Also, the chemokines include chemerin, resistin, apelin, visfatin, leptin, etc. [52].

The secretory factors play key functions in the metabolism, pathophysiology conditions including cardiovascular complications, diabetes, obesity and some cancers. Secreted factors may include many cytokines and/or chemokines such as TNF-α, IL-6, -8, as well as leptin, myostatin, and adiponectin. These secreted factors are secreted by not only adipose tissues but also present in the other tissues/cells including macrophages and muscle cells etc. [53]. In particular, the WAT are major endocrine organ and it is increasingly cross linked with muscle tissue in term of energy homeostasis and maintain the blood glucose level in the body. The adipocytes and myocytes cells secrete a broad range of bioactive proteins. In general, the adipocytes secretory proteins are termed as adipokines and myokines for muscle cells. Myokines and adipokine are important secretory molecules to be involved in local autocrine/paracrine interactions within muscle and adipose tissue respectively. There are some similar secretory proteins were identified between the myokines and adipokines, in that both groups can produce some commonality inflammation related secretory proteins, for example IL-6, Il-8 and MCP-1 (Monocyte Chemoattractant Protein-1). Trayhurn et al. [54] studied the IL-6 secreted from muscle could enhance the lipolysis in adipose tissue, whereas adipocyte derived IL-6 may induce insulin resistance in muscle cells.

In addition, the adipogenic factors include glucocorticoids, PPAR-γ agonists, insulin, and basic human fibroblast growth factor (hFGF-B) are often found that to induce cellular network assembly and/or the communication of muscle and macrophage cells [55]. Recently, Cui et al. [56] reported the communication between muscle cell and adipocytes of chicken using a trans-well co-culture chamber. After co-culture, the MSCs in the proliferation stage (20% confluence) was inhibited the differentiation of intramuscular preadipocytes (IMPs). On the other hand, the Muscle satellite cells (MSCs) in the stationary phase growth (100% confluence) would certainly accelerate the differentiation of IMPs. In addition, the gene expression levels of PPARγ, LPL and ACC accelerated in the treated co-culture cells.

## 6. Models for Co-Culture System

**Figure 2:**
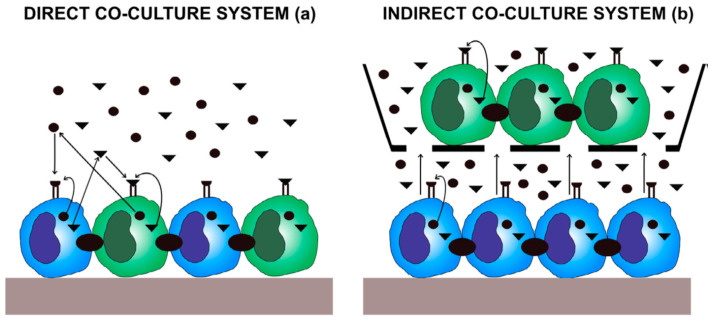
Diagrammatic representation of the cell interactions in the co-culture systems. In the direct co-culture model (**a**) cell–cell communication occurs through direct cell contact, while (**b**) indirect cell contact is communicated by autocrine and paracrine approaches (adapted from Borciani et al. (2020) [63]).

Muscle-adipocyte interactions involve a complex set of signaling events that act at multiple levels in the developmental process [57]. Skeletal muscle and fat both originate from mesenchymal stem cell precursors. That adipocytes and muscle cells have similar origins might suggest a strong degree of communication between these two cell types (Figure 2). On the other hand, muscle influences energy metabolism and inflammatory conditions in the whole body through active metabolism of fuel. During these phenomena, muscle metabolites and secretory molecules communicate with adipose and other tissues in a complex manner. Physical activity may reduce fat deposition through secretion of beneficial myokines from muscle tissue and increase insulin sensitivity and muscle mass [58]. Adipose tissue may be affected by muscle inflammation. De Boer et al. (2014) [59] studied the cross-talk between adipocytes and macrophages in an obese adipose tissue model. The pro-inflammatory adipokine profile develops through adipocyte-macrophage cross-talk and leads to decreased insulin sensitivity within adipocytes as well as in other metabolic organs, such as skeletal muscle and liver. In addition, dysfunctional adipose tissue secretes distress signaling molecules, such as chemokines and free fatty acids, which induce the pro-inflammatory cytokine production that characterizes obese adipose tissue.

Recently, co-culture techniques have been used to examine the importance of (in)direct contact between two cell types, such as muscle cells and adipocytes [60,61]. Seo et al. (2019) [22] found that muscle cell growth is disturbed by adipocytes and dominate the culture muscle cells when they are co-cultured either muscle or adipocyte cell culture. Adipocytes may communicate with myocytes to inhibit muscle cell differentiation through paracrine signaling. Mouse 3T3-L1 adipocytes attenuate the differentiation of C2C12 skeletal muscle cells by downregulating myogenin gene expression and upregulating that of myostatin, atrogin-1, and MuRF-1. Also, 3T3-L1 adipocytes induce secretion of IL-6 in C2C12 muscle cells. This area of research requires further investigation, including research into the beneficial effects of individual myokines and the mechanisms underlying muscle-adipose tissue interactions that could be employed to develop new drugs to treat chronic metabolic diseases. Also, it is important to carry out clinical studies in order to translate animal data into novel therapeutic approaches to human system [46,62].

### 6.1. Direct Co-Culture Models

Direct co-culture systems may vary in the conditions of intercellular interactions, which include cell–cell and/or cell–matrix interactions, release of secretory factors, and a combination of the above [64]. Direct physical contact promotes cell–cell interactions through surface proteins that mimics the actual in vivo situation and may increase transduction of cellular signals between various types of cells [65]. A direct co-culture model may produce different results depending on the number of cells that are seeded and the nature of the scaffolds that are used. Thus, some authors attempted to determine whether secreted factors alone, or a combination of secreted factors and other modes of cell–cell communication, play a predominant role, by controlling a single variable [66]. However, physical contact between cells may play a role in chondro-induction as compared with soluble factors alone, and paracrine factors were not shown to be involved in the expression of chondrogenic genes [67].

### 6.2. Indirect Co-Culture Models

Co-culture systems with culture inserts can be used to study cell–cell interactions under normal, differentiation, and special development environments [68]. Indirect contact co-culture models physically separate the different cell types using a trans-well chamber, membrane inserts, and/or a micro-patterned co-culture set up [69]. Two- and three-dimensional co-culture systems have been used to examine the secretome of obese adipocytes and to show that it negatively affects the contractile complex of myocytes; this represents an important advancement in our understanding of adipocyte–myocyte interactions in metabolic disease states [70]. Notably, co-culture systems are used to study the mechanisms of two-way communication between two different cell populations, in which different paracrine factors are secreted by both cell types and equally affect the two cell types. Secretory effects can be investigated using trans-well porous membrane inserts that separate different populations of cells in co-culture plates/discs (Figure 2). Importantly, indirect co-culture has been used to determine the importance of trophic factor secretion in cellular differentiation-related processes [71].

Cells grown in an insert can be co-cultured in a culture dish containing a different cell type to assess cellular communication through paracrine signaling in the absence of physical contact between cells. The insert co-culture system provides various benefits over other co-culture models, i.e., bidirectional signaling, population-specific detection of cellular changes, conservation of cell polarity, and so on [72]. Moreover, co-culture techniques can be utilized in cancer, angiogenesis, inflammation and cell differentiation studies. These co-culture systems are most important for the study of the complex cellular communications that exist between different cell types—including nerve, muscle, adipose, and immune cells—particularly in the contexts of inflammation, regulation of fat deposition and muscle development. In addition, depending on the co-culture set-up, the cell populations can be perfectly mixed or partially separated using membrane inserts containing 0.45 µM pores. Generally, co-culture with inserts is used to divide the cell populations using permeable membranes to control population interactions, which can be the main factor in achieving a stable cell culture system [73]. Separation of two different cell populations needs to be done carefully to ensure that the environment is relevant to the primary aim of the co-culture experiments. For example, if two cellular populations are dependent on each other for exchange of substances, the permeability of the materials must be considered, given that diffusion rates within specific ranges may be required. When diffusion rates are too low, important nutrients cannot be exchanged between cells. Also, a diffusion rate that is too high may spoil the whole system [74]. Therefore, it is necessary to take these factors into consideration when expanding co-culture systems to greater volumes, as diffusion is a distance-dependent phenomenon [40].

Recently, Saldana et al. (2017) [75] studied co-cultures of MSC and immune cells using a cell culture insert consisting of a polyester membrane with a 0.4 μm pore size that allows endocrine contact between the two cell types in the absence of direct cell–cell contact. Similarly, Nitta et al. (2013) [29] reported on a co-culture of activated splenocytes and adipocytes without direct cell–cell contact and showed that the co-cultured cells increased secretion of TNF-α in a time-dependent manner that reached a maximum at 20 h. Co-cultured splenocytes and adipocytes can communicate via cell surface molecules, which can in turn activate intracellular signaling pathways via TNF-α receptor signaling cascades.

## 7. Conclusions

Recently, co-culture systems of myogenic and adipogenic cells have been used to explore several important phenomena, including whether secretory factors released by the cells alter the viability and development of pre-adipocytes into mature adipocytes. Paracrine factors may influence the activity of these co-cultured cells, and significant differences exist between individual strains of muscle satellite cells and pre-adipocytes. Continued modification and use of this co-culture model provides a fuller description of the in vivo environment than is possible with the use of single in vitro cultures. This system will prove valuable in elucidating the intracellular communication that is necessary for growth and development of muscle and adipose tissue under different conditions.

## Data Availability

Not Applicable.
